# Recent Stressful Life Events in Euthymic Major Depressive Disorder Patients: Sociodemographic and Clinical Characteristics

**DOI:** 10.3389/fpsyt.2020.566017

**Published:** 2020-09-11

**Authors:** Gianluca Serafini, Xenia Gonda, Giovanna Canepa, Pierre A. Geoffroy, Maurizio Pompili, Mario Amore

**Affiliations:** ^1^Section of Psychiatry, Department of Neuroscience, Rehabilitation, Ophthalmology, Genetics, Maternal and Child Health, University of Genoa, Genoa, Italy; ^2^IRCCS Ospedale Policlinico San Martino, Genoa, Italy; ^3^Department of Psychiatry and Psychotherapy, St. Rokus Clinical Center, Semmelweis University, Budapest, Hungary; ^4^MTA-SE Neuropsychopharmacology Research Group, Hungarian Academy of Sciences and Semmelweis University, Budapest, Hungary; ^5^NAP-2-SE New Antidepressant Target Research Group, Hungarian Brain Research Programme, Semmelweis University, Budapest, Hungary; ^6^Department of Psychiatry and Addiction Medicine, AP-HP, Hopital Bichat-Claude Bernard, Paris, France; ^7^NeuroDiderot, Inserm, Paris University, Paris, France; ^8^Department of Neurosciences, Suicide Prevention Center, Sant’Andrea Hospital, University of Rome, Rome, Italy

**Keywords:** negative distressing/stressful life events, family history of suicide, major depressive disorder, previous psychiatric medications, antidepressant medications

## Abstract

**Background:**

Stressful life events (SLE) may influence the illness course and outcome. This study aimed to characterize socio-demographic and clinical features of euthymic major depressive disorder (MDD) outpatients with SLE compared with those without.

**Methods:**

The present sample included 628 (mean age=55.1 ± 16.1) currently euthymic MDD outpatients of whom 250 (39.8%) reported SLE and 378 (60.2%) did not.

**Results:**

After univariate analyses, outpatients with SLE were most frequently widowed and lived predominantly with friends/others. Moreover, relative to outpatients without SLE, those with SLE were more likely to have a family history of suicidal behavior, manifested melancholic features, report a higher Coping Orientation to the Problems Experienced (COPE) positive reinterpretation/growth and less likely to have a comorbid panic disorder, residual interepisodic symptoms, use previous psychiatric medications, and currently use of antidepressants. Having a family history of suicide (OR=9.697; *p*=≤.05), history of psychotropic medications use (OR=2.888; *p*=≤.05), and reduced use of antidepressants (OR=.321; *p*=.001) were significantly associated with SLE after regression analyses. Mediation analyses showed that the association between current use of antidepressants and SLE was mediated by previous psychiatric medications.

**Conclusion:**

Having a family history of suicide, history of psychotropic medications use, and reduced use of antidepressants is linked to a specific “at risk” profile characterized by the enhanced vulnerability to experience SLE.

## Background

Stressful life events (SLE) may be defined as: “environmental events or chronic conditions that objectively threaten the physical and/or psychological health or well-being of individuals of a particular age in a particular society” (1). Life events may be described as social and environmental occurrences leading to psychophysiological modification in the general population during the time course (2). Life events are considered positive or negative according to the individual’s subjective experience. Negative life events, which may have a prominent role in the development and clinical course of psychiatric disorders may be identified in various social/environmental contexts (2) and may be commonly defined as: 1) maltreatment and violence; 2) loss events; 3) intrafamilial problems; 4) school and interpersonal problems (3, 4). These type of life events are usually associated with undesirable affective experiences exerting deleterious effects on the individual mental health and social/environmental adaption (4). Generally, SLE such as perceived loss, humiliation, entrapment, and perceived danger seem to provide interesting information about the course and outcome of psychiatric conditions (5). Notably, certain types of SLE such as involuntary occupation loss and marital separation may enhance suicide risk (6, 7), and influence the time to remission of major depressive disorder (MDD) (8) as well as substance use (9).

Based on stress vulnerability models, having experienced SLE is generally linked to poor mental health (10) with the development of major psychiatric conditions generally associated with the exposure to SLE (11). Having experienced more proximal life personal and situational factors may also enhance suicide risk (12) with the cumulative effect of multiple life events playing a more relevant effect than that of specific single life events (13).

While depression has a moderate heritability, environmental events account for approximately 60% of the variation in depression (14) on a general population level depending on the severity of the illness, with 80% of depressive episodes preceded by a major stress exposure (15) and etiologically relevant distal and proximal stressors occurring quite frequently, about at least once every 3–4 years.

SLE are able to strongly predict both the onset (16) and recurrence (17) of depressive episodes, including suicidal behavior. Evidence also suggested that negative life adversities are important risk factors particularly for depressed subjects with a negative cognitive style (18). This hypothesis postulated that exposure to SLE may determine sensitization or kindling and may enhance the individual vulnerability to manifest depressive episodes, although the patient is not directly experiencing a relevant psychosocial stressor. Socio-demographic and clinical variables such as gender potentially mediate the relation between SLE and depression. For instance, when compared to males, females seem to be more vulnerable to the adverse effects of psychosocial stressors (19). Studies (19, 20) stressed the link between the personality trait “neuroticism,” which increases the likelihood of maladaptive responses and coping in the face of negative life events and increased sensitivity to stress and severity of MDD. Importantly, Kendler and colleagues (20) found that, in a sample of more than 7500 twins, higher neuroticism enhanced the likelihood of depressive episodes in response to SLE.

The above indicates that SLE should be taken into consideration in affective disorder patients due to their impact on illness course and outcomes including suicide. Thus, the present study aimed to compare socio-demographic/clinical characteristics (including marital and living level, family history of psychiatric conditions and/or suicidal behavior, melancholic characteristics in the past, residual interepisodic symptoms, psychiatric medications in the past, current antidepressant medications use, and specific psychometric measures) of euthymic MDD outpatients experiencing significant SLE in the previous 6 months and those who did not, focusing on specific features that may explain the differential burden of disease directly linked to having or not experienced recent SLE. Our aim was also to test whether the effect of current antidepressants use on SLE was mediated by specific variables (i.e., previous psychiatric medications, family history of suicide). Here, we mainly hypothesize that previous psychiatric medications have a significant direct/indirect effect on SLE.

## Methods

### Sample

The present sample is composed of 628 currently euthymic MDD outpatients with age ranging from 18 to 85 years (mean age=55.1 ± 16.1). Clinically, euthymia was defined using a Montgomery–Asberg Depression Rating Scale (MADRS) (21) score of <10. Our sample included predominantly single MDD outpatients (52.9%) and, in a relatively smaller proportion, recurrent MDD outpatients (47.1%). Participants are all consecutive euthymic unipolar outpatients receiving only maintenance treatment at the time of evaluation. Overall, 488 subjects (77.7%) of our sample were treated with antidepressant medications and 140 individuals (22.3%) received only psychotherapy as maintenance. In particular, psychopharmacological treatments and psychopathological conditions were stable for at least 6 months before assessment. All euthymic MDD outpatients were recruited from our catchment area and have been followed/treated by our university outpatient service for at least 12 months. In addition, they were regularly followed by our local psychiatric services.

### Measures and Study Design

An investigator (GC) who was adequately trained to improve the interrater reliability administered to participants the following psychometric instruments: 1) Clinical Global Impression (CGI) (22); 2) Beck Hopelessness Scale (BHS) (23); 3) Hamilton Anxiety Rating Scale (HARS) (24); 4) Global Assessment of Functioning Scale (GAF) (American Psychiatric Association, 1987) (25); 5) Childhood Trauma Questionnaire (CTQ) (26); 6) Coping Orientation to the Problems Experienced (COPE) (27); 7) Toronto Alexithymia Scale (TAS-20) (28) to collect other relevant clinical information.

All clinical information were retraced, as specified, by using clinical files and lifetime computerized medical records. All outpatients accepted voluntarily to participate in the present study and provided their informed consent to participate. The local Ethical Review Board regularly approved the specified study design.

### Ascertainment of Recent Stressful Life Events

Significant stressful/distressing life-events in the previous 6 months were collected based on self-report and described as follows: 1) maltreatment and violence (sexual/physical/emotional abuse, emotional/physical neglect, witnessing home/community aggression); 2) loss events (separations, death of a parent or close friend); 3) intrafamilial problems (parental divorce, family instability, social or economic problems); 4) school and interpersonal problems (failure of grade in school or in exam, breaking up with a close friend, and poor social relationships) (for more details, see 3 and 4).

### Procedure and Data Collection

All MDD outpatients were recruited at the Department of Neuroscience (DINOGMI), University of Genoa, outpatient service, between July 2014 and February 2020. We used the following inclusion criteria: 1) a diagnosis of single/recurrent MDD; 2) euthymia; 3) a current age of >18 years. Depressive symptoms related to MDD were classified based on the Diagnostic and Statistical Manual of Mental Disorders, 5^th^ edition (DSM-5) (29). First, an investigator (GC) examined clinical records to retrace subjective histories and clinical variables; then, two senior authors (GS, MA) systematically and independently verified data by using the Mini International Neuropsychiatric Interview (MINI) (30) updated to map to DSM-5 (29). Direct interviews with patients/family members as well as investigation of existing medical records allow the detailed collection of clinical information regarding affective episodes prior the patients’ recruitment in our outpatient service. Specifically, we performed a comprehensive data collection using the following variables: 1) socio-demographic information; 2) individual data (e.g., personal autonomy, lifetime substance abuse/dependence, use of psychiatric medications and psychotherapy in the past); 3) positive history of clinical conditions/negative outcome in family (e.g., psychiatric disorders and suicidal behavior); 4) comorbid conditions (e.g., medical/psychiatric disorders); 5) illness course features (e.g., manifested melancholic features and/or current atypical depressive characteristics, seasonality, psychotic symptoms at first episode, single/recurrent episode, residual interepisodic symptoms, illness duration and untreated illness, current illness episode, number of previous affective episodes, age of illness onset, age at first treatment and first hospitalization, and lifetime suicide attempts).

Exclusion criteria were as follows: 1) all clinical conditions that may influence the ability to complete the evaluations including conditions such as Alzheimer’s disease, delirium, or severe neurological disorders like intellectual disability; 3) denial of informed consent, 4) a history of active abuse/dependence (throughout the previous 6 months). Lifetime substance use emerging during mental examination, was not considered a specific exclusion criterion. Intellectual disability was investigated using the Wechsler Adult Intelligence Scale (31).

### Statistical Analyses

Subjects were categorized based on the presence/absence of SLE in the previous 6 months and divided into two groups similarly to existing published studies (32) outpatients with recent SLE and 2) outpatients without recent SLE. Data for categorical variables were analyzed using Student’s t-tests and Pearson chi-square/Fisher’s exact test in contingency tables (χ^2^). The Kolmogorov–Smirnov test was performed to confirm whether all the investigated variables in the sample followed the normal distribution. Significance was set at *P* ≤ 0.05 (two-tailed).

A binary logistic regression analysis considering specific socio-demographic characteristics (marital status that was dichotomized in married/unmarried status and living status that was dichotomized in living alone or not alone) together with other variables such as family history of suicidal behavior, comorbid panic disorder, previous psychiatric medications, manifested melancholic features in the past, interepisodic residual symptoms, current antidepressants use, and COPE positive reinterpretation and growth was carried out to investigate the contribution of these characteristics in predicting presence/absence of SLE.

Multicollinearity and extreme cases were excluded, and the normality of residuals (histograms and P–P plots) was appropriately checked.

**Figure 1:**
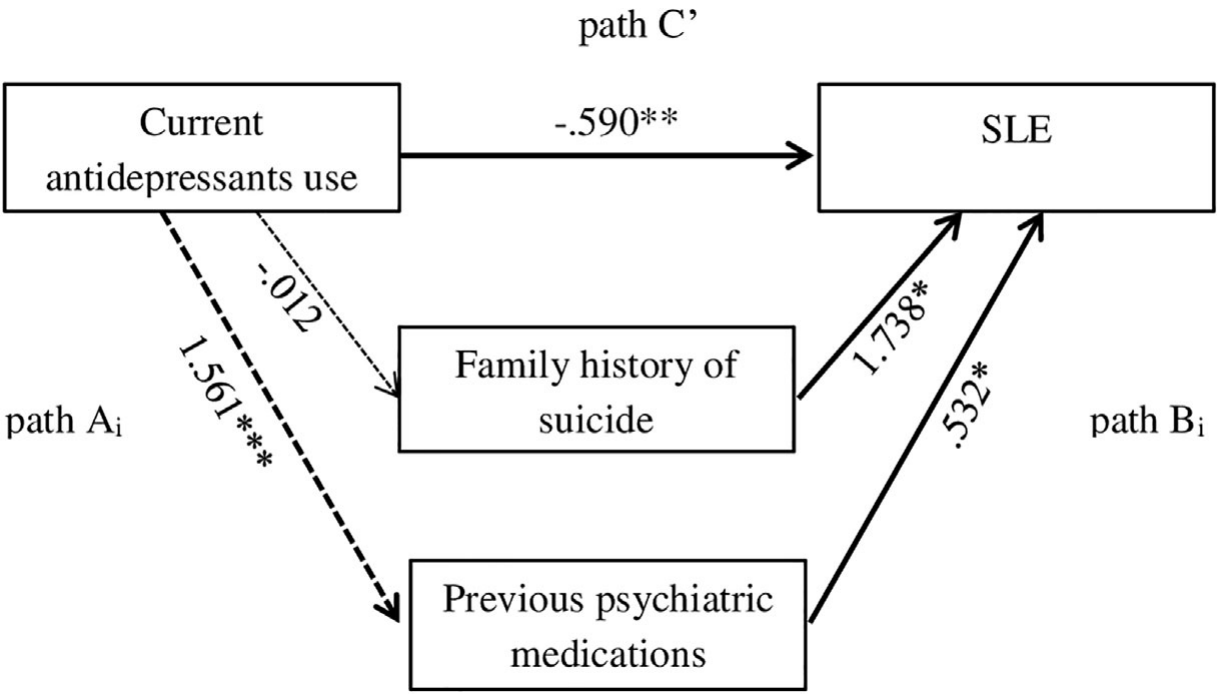
Mediation model with potential mediators (Paths Ai: Variable i→Mediator; Paths Bi: Mediator→Dependent Variable; Path C’: Independent Variable→Dependent Variable). *p = ≤ .05; **p = ≤ .01; ***p = ≤ .001.

To assess whether the effect of current antidepressants use on SLE was mediated by specific variables (i.e., previous psychiatric medications, family history of suicide), single-mediator models were tested according to the strategy recommended by Preacher and Hayes (33). In a single-mediator model, an independent variable (X = current antidepressants use) is hypothesized to act on the outcome variable (Y = SLE) in two ways: X change a mediator (e.g., Mi = previous psychiatric medications or family history of suicide; path Ai) that, in turn, changes an outcome variable (Y; path Bi), or X changes Y directly (path C′) (for more details, see **Figure 1**).

The Statistical Package for Social Sciences (SPSS) for Windows 21.0 was used to carry out all the statistical analyses.

## Results

### Socio-Demographic and Clinical Variables of Outpatients With Significant Stressful Life Events in the Previous 6 Months Compared With Those Without

The sample includes 628 euthymic MDD outpatients who were consecutively and voluntarily recruited at the outpatient service of Department of Neuroscience (DINOGMI), University of Genoa from the catchment area. Socio-demographic and clinical features of outpatients are summarized in **Table 1**.

**Table 1:** Socio-demographic and clinical variables (categorical and quantitative) in patients with significant distressing life-events in the last 6 months (N=250) compared with those without (N=378).

Variables	Subjects with significant distressing life events in the previous 6 months (*N*=250)		Subjects without significant distressing life events in the previous 6 months (*N*=378)		Statistic (*χ*^2^)	*♦p*
	*N*	%	*N*	%		
**Gender**
Male	87	36.7	150	63.3	*χ*^2^_(1)_=1.527	.125*
Female	163	41.7	228	58.3		
**Marital status**						
Single	62	33.7	122	66.3	*χ*^2^_(3)_=12.963	**.005**
Married	116	38.5	185	61.5		
Divorced	35	43.8	45	56.3		
Widowed	34	59.6	23	40.4		
**Living status**						
Alone	66	42.9	88	57.1	*χ*^2^_(3)_=9.729	**.021**
With family	163	37.0	278	63.0		
With friends	9	64.3	5	35.7		
With others	6	75.0	2	25.0		
**Working status**						
Employed	113	40.6	165	59.4	*χ*^2^_(3)_=3.635	.304
Unemployed	59	45.7	70	54.3		
Retired	65	36.5	113	63.5		
Student	8	30.8	18	69.2		
**Socio-economic level**						
Below average	76	39.6	116	60.4	*χ*^2^_(2)_=.659	.719
Average	154	40.6	225	59.4		
Above average	14	34.1	27	65.9		
**Positive history of psychiatric conditions in family**						
No	163	38.3	263	61.7	*χ*^2^_(1)_=.367	.302*
Yes	78	40.8	113	59.2		
**Family history of suicidal behavior**						
No	144	38.1	234	61.9	*χ*^2^_(1)_=5.818	.020*
Yes	7	77.8	2	22.2		
**Psychiatric comorbidity**						
No	197	40.3	292	59.7	*χ*^2^_(1)_=.077	.431*
Yes	53	39.0	83	61.0		
**Comorbid anxiety**						
No	234	39.5	359	60.5	*χ*^2^_(1)_=.747	.240*
Yes	16	47.1	18	52.9		
**Comorbid panic disorder**						
No	243	40.7	354	59.3	*χ*^2^_(1)_=3.595	.041*
Yes	7	23.3	23	76.7		
**Comorbid phobias**						
No	247	39.6	376	60.4	*χ*^2^_(1)_=2.072	.177*
Yes	3	75	1	25		
**Comorbid obsessive compulsive disorder**						
No	250	40.2	372	59.8	*χ*^2^_(1)_=3.342	.078*
Yes	0	0.0	5	100		
**Comorbid post-traumatic stress disorder**						
No	250	39.9	376	60.1	*χ*^2^_(1)_=.664	.601*
Yes	0	0.0	1	100.0		
**Comorbid personality disorder, cluster A**						
No	250	40	375	60.0	*χ*^2^_(1)_=1.331	.361*
Yes	0	0.0	2	100.0		
**Comorbid personality disorder, cluster B**						
No	241	39.7	366	60.3	*χ*^2^_(1)_=.227	.399*
Yes	9	45.0	11	55.0		
**Comorbid personality disorder, cluster C**						
No	247	39.7	375	60.3	*χ*^2^_(1)_=.852	.315*
Yes	3	60.0	2	40.0		
**Psychiatric medications in the past**						
No	36	30.0	84	70.0	*χ*^2^_(1)_=6.000	.009*
Yes	213	42.2	292	57.8		
**Non psychiatric medications in the past**						
No	121	41.0	174	59.0	*χ*^2^_(1)_=.379	.297*
Yes	127	38.6	202	61.4		
**Lifetime substance abuse/dependence**						
No	237	39.8	359	60.2	*χ*^2^_(1)_=.003	.563*
Yes	11	39.3	17	60.7		
**Psychotherapy in the past**						
No	203	39.3	313	60.7	*χ*^2^_(1)_=.002	.523*
Yes	40	39.6	61	60.4		
**Single/recurrent episode**						
Single	129	41.5	182	58.5	*χ*^2^_(1)_=.842	.203*
Recurrent	93	37.7	154	62.3		
**First depressive episode**						
Depressive	9	23.1	30	72.9	*χ*^2^_(1)_=.237	.414*
Nondepressive	9	28.1	23	71.9		
**Current catatonic characteristics**						
No	159	39.7	242	60.3	*χ*^2^_(1)_=1.962	.222*
Yes	0	0.0	3	100.0		
**Current melancholic characteristics**						
No	141	37.9	231	62.1	*χ*^2^_(1)_=6.538	.010*
Yes	19	61.3	12	38.7		
**Current atypical depressive characteristics**						
No	153	40.3	227	59.7	*χ*^2^_(1)_=.875	.239*
Yes	7	30.4	16	69.6		
**Psychotic symptoms at first episode**						
No	140	40.2	208	59.8	*χ*^2^_(1)_=1.573	.176*
Yes	6	60.0	4	40		
**BMI**						
<18	10	50.0	10	50.0	*χ*^2^_(3)_=4.977	.173
18–25	191	41.7	267	58.3		
25–30	39	32.0	83	68.0		
>30	9	34.6	17	65.4		
**Residual interepisodic symptoms**						
No	116	36.4	203	63.6	*χ*^2^_(1)_=3.024	.049*
Yes	118	43.4	154	56.6		
**Lifetime suicide attempts**						
None	240	40.3	183	92.9	*χ*^2^_(2)_=2.292	.318
0–3	7	25.9	13	6.6		
>3	1	33.3	0	0.0		
**Seasonality**						
No	144	39.0	225	61.0	*χ*^2^_(1)_=.649	.340
Yes	2	25.0	6	75.0		
**Adherence to treatment**						
Bad	11	36.7	19	63.3	*χ*^2^_(1)_=.43	.500*
Good	132	38.6	210	61.4		
**Current anxyolitic medications**						
None	96	41.6	135	58.4	*χ*^2^_(3)_=.641	.887
Short half-life	75	38.9	118	61.1		
Middle half-life	57	37.7	94	62.3		
High half-life	19	40.4	28	59.6		
**Current antidepressant medications**						
No	70	51.1	67	48.9	*χ*^2^_(1)_=9.273	.002*
Yes	179	36.7	309	63.3		
**Personal autonomy**						
Bad	18	52.9	140	38.3	*χ*^2^_(1)_=2.809	.069*
Good	16	47.1	226	61.7		
****						
	**Subjects with significant distressing life-events in the previous 6 months (*N*=250)**		**Subjects without significant distressing life-events in the previous 6 months (*N*=378)**		**Statistic (Student’s *t*-test)**	***p***
	**Mean**	**SD**	**Mean**	**SD**		
**Current age**	55.1	15.7	54.8	16.4	*T*_626_=-.605	.841
**Educational level (years)**	11.3	3.5	11.6	3.5	*T*_583_=.426	.332
**Number of illness episodes**	1.8	1.4	1.9	1.5	*T*_499 =_ 1.202	.549
**Age at first treatment**	47.8	16.7	45.2	16.6	*T*_550_=.055	.084
**Duration of untreated illness (years)**	3.7	26.4	1.7	3.3	*T*_468 =_ 4.582	.223
**Duration of illness (years)**	9.6	12.9	11.8	14.2	*T*_528 =_ 2.455	.070
**Duration of substance abuse (years)**	0.4	2.5	0.4	2.4	*T*_609_=-.017	.962
**MADRS total score**	6.5	3.3	6.2	3.5	*T*_203 =_ 1.686	.530
**CGI severity (item 1)**	2.1	4.1	1.4	2.5	*T*_174 =_ 5.844	.139
**BHS total score**	10.7	6.2	11.03	5.8	*T*_429_=.619	.545
**HARS total score**	7.9	8.4	8.2	7.5	*T*_174_=.864	.791
**GAF Mental Composite Score**	11.1	2.5	11.6	4.2	*T*_463_=.560	.161
**GAF Physical Composite Score**	14.9	1.9	14.8	1.8	*T*_450 =_ 1.732	.790
**CTQ emotional abuse**	3.2	5.03	3.3	4.7	*T*_422_=.149	.842
**CTQ physical abuse**	1.5	3.2	1.6	4.01	*T*_440_=.303	.800
**CTQ sexual abuse**	1.7	4.1	1.2	3.5	*T*_431 =_ 7.205	.177
**CTQ emotional neglect**	13.1	7.1	14.04	6.7	*T*_425_=.775	.188
**CTQ physical neglect**	5.3	3.4	5.2	3.5	*T*_119_=.311	.917
**COPE active**	11.2	5.9	10.3	2.5	*T*_457_=.9422	**.036**
**COPE planning**	10.6	4.6	10.5	3.6	*T*_445_=.908	.877
**COPE suppression**	10.1	5.1	9.9	3.4	*T*_453 =_ 2.102	.643
**COPE behavioral disengagement**	8.8	3.6	9.1	4.2	*T*_449_=.101	.477
**COPE denial**	7.7	4.4	7.3	2.4	*T*_450 =_ 3.111	.147
**COPE mental disengagement**	8.9	2.6	9.1	4.2	*T*_446_=.082	.553
**COPE substance abuse**	5.02	2.3	5.3	3.1	*T*_460 =_ 2.211	.286
**COPE emotional social support**	4.4	72.6	9.7	3.9	*T*_454 =_ 2.983	.234
**COPE instrumental social support**	10.5	4.6	10.3	3.6	*T*_466_=.699	.727
**COPE venting emotions**	10.7	4.7	10.2	3.3	*T*_443 =_ 1.384	.138
**COPE positive reinterpretations**	10.1	4.3	10.2	3.8	*T*_468_=.603	.904
**COPE restraint**	9.7	5.1	10.3	4.8	*T*_445_=.066	.188
**COPE acceptance**	9.9	3.6	10.1	4.8	*T*_447_=.048	.652
**COPE turning to religion**	8.8	5.3	8.6	4.5	*T*_460_=.142	.670
**COPE humor**	6.7	2.9	6.7	3.02	*T*_433_=.045	.879
**TAS total score**	63.2	13.6	63.8	18.6	*T*_370 =_ 1.237	.710
**TAS difficulties in identifying feelings**	17.4	7.1	17.5	8.9	*T*_419_=.090	.874
**TAS difficulties in communicating feelings to others**	15.3	4.4	15.4	5.7	*T*_425_=.020	.833
**TAS thoughts oriented to external context**	27.7	5.4	28.6	9.2	*T*_431 =_ 3.091	.246
**DAI total score**	12.9	4.3	14.03	4.8	*T*_277_=.897	.066

BHS, Beck Hopelessness Scale; COPE, Coping Orientation to the Problems Experienced; CTQ, Childhood Trauma Questionnaire; DAI, Drug Attitude Inventory; HARS, Hamilton Anxiety Rating Scale; MADRS, Montgomery-Asberg Depression Rating Scale; TAS, Toronto Alexithymia Scale; *Fisher’s exact test (significance two-tailed); percentages were calculated per column.

### Frequency of Recent Stressful Life Events and Associated Socio-Demographic and Clinical Characteristics

Among subjects who were recruited, 250 (39.8%) reported significant SLE in the previous 6 months and 378 (60.2%) did not.

Those reporting recent SLE [87 males (36.7%) and 163 females (41.7%)] differed from individuals who did not report recent SLE. Specifically, when compared with those who did not report recent SLE subjects with recent SLE differed in terms of both marital and living status (χ^2^_(3)_=12.963, *p*=.005; χ^2^_(3)_=9.729, *p*=≤.05), respectively.

Relative to outpatients without recent stressful life events, those who have experienced these events were more likely to have a family history of suicidal behavior (77.8 *vs.* 22.2%) (χ^2^_(1)_=5.818, *p*=≤.05), less likely to have a comorbid panic disorder (23.3 *vs.* 76.7%) (χ^2^_(1)_=3.595, *p*=≤.05) and less likely to have used psychiatric medications (42.2 *vs.* 57.8%) (χ^2^_(1)_=6.000, *p*=≤.01).

When compared to subjects without SLE in the previous 6 months, subjects with recent stressful events were also more likely to have exhibited melancholic characteristics in the past (61.3 *vs.* 38.7%) (χ^2^_(1)_=6.538, *p*=.01) and less likely to manifest residual interepisodic symptoms (43.4 *vs.* 56.6%) (χ^2^_(1)_=3.024, *p*=≤.05) and use current antidepressant medications (36.7 *vs.* 63.3%) (χ^2^_(1)_=9.273, *p*=≤.005).

The Kolmogorov–Smirnov test did not find any significant abnormality concerning the continuous data. Moreover, subjects with recent SLE were more likely to have a higher COPE positive reinterpretation and growth (11.2 ± 5.9 *vs.* 10.3 ± 2.5, t_457 =_ 9.422, *p* ≤.05) when compared to those without SLE in the previous 6 months (for more details, see **Tables 1** and **2**).

**Table 2:** Medical comorbidity in patients with significant distressing life-events in the previous 6 months (N=250) compared with those without (N=378).

	Subjects with significant distressing life-events in the previous 6 months (*N*=250)		Subjects without significant distressing life-events in the previous 6 months (*N*=378)		Statistic (*χ*^2^)	*p*
	*N*	%	*N*	%		
**Medical comorbidities**
No	143	65.0	77	35.0	*χ*^2^_(1)_=3.193	.064*
Yes	233	57.7	171	42.3		
**Neurological comorbid disorders**						
No	358	60.6	233	39.4	*χ*^2^_(1)_= .475	.304*
Yes	18	54.5	15	45.5		
**Cardiological comorbid disorders**						
No	175	89.3	170	95.0	*χ*^2^_(1)_=.001	.555*
Yes	21	10.7	9	5.0		
**Endocrinological comorbid disorders**						
No	341	59.5	232	40.5	*χ*^2^_(1)_=1.625	.130*
Yes	35	68.6	16	31.4		
**Inflammatory/immunological comorbid disorders**						
No	342	59.8	230	40.2	*χ*^2^_(1)_=.623	.262*
Yes	34	65.4	18	34.6		
**Metabolic disorders**						
No	366	60.4	240	39.6	*χ*^2^_(1)_=.171	.427*
Yes	10	55.6	8	44.4		
**Mild cognitive impairment (amnesic)**						
No	196	100.0	178	99.4	*χ*^2^_(1)_=1.098	.477*
Yes	0	0.0	1	0.6		
**Comorbid polmonary diseases**						
No	194	99.0	179	100.0	*χ*^2^_(1)_=1.836	.273*
Yes	2	1.0	0	0.0		
**Comorbid cancer**						
No	189	96.4	169	94.4	*χ*^2^_(1)_= .878	.246*
Yes	7	3.6	10	5.6		
**Comorbid chronic renal failure**						
No	196	100.0	178	99.4	*χ*^2^_(1)_= 1.098	.477*
Yes	0	0.0	1	0.6		
**Comorbid HIV**						
No	194	99.0	179	100.0	*χ*^2^_(1)_=1.836	.273*
Yes	1	1.0	1	0.0		

*HIV, human immunodeficiency virus; Fisher’s exact test (significance two-tailed); percentages were calculated per column.

### Multivariate Regression Analyses Including Stressful Life Events as Dependent Variable in the Total Sample

All clinical variables which were significant at the univariate analyses were entered into a binary logistic regression analysis with SLE as dependent variable in order to identify independent factors which resulted associated with. Specific socio-demographic characteristics such as marital and living status as well as clinical variables (e.g., family history of suicidal behavior, comorbid panic disorder, previous psychiatric medications, manifested melancholic features in the past, interepisodic residual symptoms, current antidepressants use, and COPE positive reinterpretation and growth) were introduced into the regression model. The amount of variation in the first dependent variable (presence/absence of recent SLE) that was accounted for all predictors (R^2^-value) was 18.3% (*p*≤.001) (see **Table 3**).

**Table 3:** Multiple regression model of significant distressing life-events in the previous 6 months in the total sample (N=628).

Variable	*B*	*SE*	Wald	P	OR	95% Cl OR	
						Lower	Upper
Married status	.021	.276	.006	.940	1.021	.595	1.754
Living status	−.010	.010	1.136	.286	.990	.971	1.009
Family history of suicide	2.272	1.146	3.931	.047	9.697	1.026	91.629
Comorbid panic disorders	−1.022	.681	2.248	.134	.360	.095	1.369
Previous psychiatric medications	1.061	.406	6.808	.009	2.888	1.302	6.407
Melancholic features in the past	.864	.592	2.129	.145	2.373	.743	7.576
Interepisodic residual symptoms	.391	.290	1.819	.177	1.479	.838	2.610
Current antidepressants use	−1.136	.349	10.627	.001	.321	.162	.636
COPE active	.065	.037	3.054	.081	1.067	9.92	1.147
Constant	−2.814	.910	9.572	.002	.060		

Dependent variable: SLE. All predictors were entered in one block (hierarchical method). Model summary: R=.113, R^2^=.153; significance: P=≤.001. Bold values denote statistical significance.

Having a family history of suicide with an OR of 9.697 (*p*=≤.05), history of psychotropic medications use with an OR of 2.888 (*p*=≤.01), and reduced use of antidepressant drugs with an OR of .321 (*p*=.001) were all significantly associated with recent SLE.

### Mediation Analyses

In a regression model with current antidepressants use and family history of suicide as predictors and SLE as criterion, the regression models were both significant (R^2^ = .020, *B* = -.590, SE=.195, Wald = 9.145, p = .002, OR = .554) (R^2^ = .020, *B* = 1.738, SE=.809, Wald = 4.620, p = .032, OR = 5.687), respectively. When considering the effect of current antidepressants use on SLE in a single mediator model (considering first family history of suicide and later previous psychiatric medications as possible mediators), the results were as follows.

Family history of suicide had a significant direct effect on SLE (family history of suicide: omnibus test of direct effect of Mi on Y: R^2^ = .020, *B* = 1.738, SE=.809, Wald = 4.620, p = .032, OR = 5.687) but a nonsignificant indirect effect (family history of suicide: omnibus test of direct effect of X on Mi: R^2^ = .000, *B* = -.012, SE=.811, Wald = .000, p = .988, OR = .988). Previous psychiatric medications had a significant direct effect on SLE (previous psychiatric medications: omnibus test of direct effect of Mi on Y: R^2^ = .013, *B* = .532, SE=.219, Wald = 5.917, p = .015, OR = 1.702) and a significant indirect effect (previous psychiatric medications: omnibus test of direct effect of X on Mi: R^2^ = .121, *B* = 1.561, SE=.219, Wald = 50.777, p = <.001, OR = 4.765).

## Discussion

Our study investigating the association of recent SLE in euthymic depressive patients indicated that the experience of recent stressful events was associated with several clinical, course, and sociodemographic characteristics, some of which may be clinically utilizable in understanding and managing the culminating effect of life stressors in influencing illness course and outcome.

### Socio-Demographic Characteristics of Outpatients With Significant Stressful Life Events in the Previous 6 Months Compared With Those Without

Euthymic outpatients who experienced SLE differed from those who did not experience SLE in terms of both marital and living status.

Existing evidence suggested that being widowed is generally perceived as one of the most SLE (34) linked to relevant impairments in the general well-being of the bereaved spouse. According to previously published studies (35), widowers and widows should be considered at higher risk to experience lower subjective well-being even several years after the death of the spouse. In line with these findings, Carnelley and colleagues (36) found that widowers/widows experienced a significant distress and reduced subjective wellbeing even decades after the death. Similarly, we found a higher ratio of those widowed among those experiencing recent distressing events.

On the other hand, conversely to existing evidence suggesting that subjects who have more contact with friends and family are happier than those who have less contact (37), we found that outpatients who experienced SLE lived predominantly with friends or others. However, living with family rather than living with friends may be generally interpreted as a protective factor for MDD patients. This discrepancy could be related, in our opinion, to the fact that, although being recruited in stable psychopathological conditions, participants of the present study are predominantly affected by MDD (single episode) which is a condition for which many of them might have searched a significant social contact or asked support due to their illness.

### Clinical Characteristics of Euthymic Outpatients Experiencing Significant Stressful Life Events in the Previous 6 Months Compared With Those Without

As for clinical variables, outpatients experiencing significant SLE were more likely to have a family history of suicidal behavior, manifested melancholic features in the past, report a higher COPE positive reinterpretation and growth, and less likely to have a comorbid panic disorder, residual interepisodic symptoms, past psychiatric medication use, and current antidepressant medication use.

In line with previous evidence (38) suggesting that the occurrence of SLE was higher and the associated perceived experience was more deleterious in suicidal patients, we found an association between SLE in the previous six months and family history of suicidal behavior. Specifically, this could be explained by the assumption that the interpersonal pattern linked to family history of suicide may consistently influence the ability to maintain stable and significant relations with others as supported by Rajalin and colleagues (39).

Moreover, we found that subjects with recent SLE were more likely to have manifested melancholic features in the past. Existing studies analyzed the association between depression subtypes and the likelihood to experience life adverisities. For instance, Kohn and colleagues (40) reported that, in a sample of 115 MDD patients, subjects with nonmelancholic depression or first depressive episode more frequently experienced ≥3 life adversities when compared to severe, melancholic, or recurrent depression. However, they investigated only a subset of MDD patients which is more prone to suffer from a cluster of negative life events. In addition, while the study of Kohn and colleagues (40) analyzed a sample of patients with mild-moderate depression, we investigated a larger (N=628) sample of MDD euthymic subjects. Thus, the main differences between our study and the study of Kohn and colleagues (40) may be related to the different study sample selection and different clinical characteristics.

Finally, we found that those who experienced SLE were more likely to report higher COPE positive reinterpretation and growth. This may be explained by the fact that the attitudes to cope with stress in our MDD patients, the psychopathological conditions of which were stable for at least 6 months before assessment, were more pronounced than those of other patient populations. Clinicians and health professionals should take appropriately into account these data in order to conduct psychotherapeutic interventions which are directed toward further coping with stress and create treatment strategies which may result especially useful in improving functional capacity in the identified stable phases of MDD.

### Predictors of Stressful Life Events and Mediators of the Associations With Stressful Life Events

After binary regression analysis, MDD patients with recent SLE were more likely to have a family history of suicide (OR=9.68) and a history of psychotropic medications use (OR=2.89), but they were less likely to use currently antidepressants (OR=.32). In particular, outpatients who have experienced recent SLE were more likely to report a family history of suicide, more likely to have used previous psychiatric medications, and less likely to currently use antidepressants drugs that those without recent SLE in the previous 6 months.

Generally, having a family history of suicidal behavior is also associated with a greater propensity to SLE; thus, clinicians should be warned about carefully searching for suicidal thoughts in this patient subgroup as these individuals might exhibit a worse combination of biological, psychological, and environmental risk for suicide. In particular, in individuals with family history of suicidal behavior, the increased likelihood to experience stressful life events may enhance the perception of fewer reasons for living, thus elevating the risk of suicidal behavior, although in our study we did not observe significant differences in terms of suicide attempts between those with and without recent stressful life events. Consistent with this hypothesis, evidence documented that having a family history of suicide or attempted suicide is a significant risk factor for suicidal behavior and supposed the existence of a specific genetic component of suicidal behavior (41, 42). In line with these results, Rajalin and colleagues (39), recently found in a sample of 181 suicide attempters that having a family history of suicide was linked to factor 2 (nonassertive; exploitable; overly nurturant, and intrusive), in particular with Intrusive scale scores of the Inventory of Interpersonal Problems (IIP). Subjects with higher scores in the Intrusive subscale of the IIP are described to be excessively controlling, not careful to respect individual and other´s boundaries as well as more prone to take charge of other subjects’ problems (39). This may imply that these individuals pervasively experience disappointment and presumably reject in social contexts appearing unable to identify themselves in positive arguments with others. According to these findings, subjects who have experienced recent SLE and report a positive history of suicide among family members seem to exhibit specific deficits in interpersonal functioning. Moreover, evidence (43) clearly suggested that having a family history of suicide may be considered not only as a biological but also a psychological risk factor for familial transmission of suicide. Unfortunately, in our study we were not able to investigate the impact of interpersonal difficulties in subjects with family history of suicidal behavior who have also experienced recent SLE. In addition to the identified at risk profile, the participants of our study were also more likely to be widowed and have used psychiatric medications in the past.

Surprisingly, after multivariate analysis, subjects with significant SLE were less likely to currently use antidepressant drugs compared to those without recent stressful life events. This finding may be presumably explained by the fact that the majority of our study participants suffer predominantly from reactive and situational depression, but it is also possible that individuals who experienced recent SLE in our study may rely on more resilient social groups in which they received social support even in the context of negative interpersonal events or may have benefited from a psychotherapeutic approach. Such support may have helped them to better cope with SLE, thus reducing the current use of antidepressant medications. However, most patients do not frequently consult psychiatrists or mental health professionals due to stigma and discrimination as well as fear of disability and chronicity related to psychiatric conditions. This may, at least partially, justify the reduced or absent psychiatric services attendance in the past.

Finally, according to mediation analyses the association between the reduced use of antidepressant drugs and SLE was mediated by the history of psychotropic medications use. Thus, with this result we confirm the initial hypothesis based on which previous psychiatric medications had a significant direct/indirect effect on SLE. Specifically, subjects with a reduced use of antidepressants may be more vulnerable to experience SLE, in particular whether they have been treated in the past with psychiatric medications (that may have been prematurely discontinued by patients). This result is in line with the questioned long-term partial benefits of psychiatric medications (44) and may reflect, in our opinion, the limited potential of these compounds, which are detrimentally interrupted by patients on their own initiative (45, 46), on protecting against long-term exposure to stressors.

Unfortunately, whether the use of pharmacological medications is not combined with other available treatment strategies (e.g., psychotherapy), depressed individuals who have been treated only with psychoactive agents may be insufficiently protected in the long-term period towards potential illness recurrences (47, 48) neither they seem to have developed specific coping strategies to successfully cope with psychosocial distress in daily life. To further confirm our findings, it has been reported that the current use of antidepressants may help subjects to face intense emotional stimuli and stressful cues (49), presumably by attenuating the brain expression and/or actions of pro-inflammatory mediators. Here, we add that having undergone a psychiatric treatment (that may have been prematurely discontinued) is associated with the more frequent occurrence of SLE, if MDD patients are not adequately treated with antidepressant medications.

## Limitations of the Study

The present study should be considered in the light of the following limitations/shortcomings. First, SLE were not measured using specific psychometric instruments such as the Social Readjustment Rating Scale (SRRS) which assesses the frequency of 43 common stressful life events occurring over the previous 12 months (34), that would allow us to rate the impact of specific stressful life experiences on a specific severity scale. In addition, we were also unable to use specific psychometric tools like the Karolinska Self Harm History Interview (50), including specific questions regarding family history of suicidal behavior in order to investigate the family history of completed suicide in a more detailed manner. In addition, the cross-sectional nature of the present study potentially leading to type I errors needs to be considered as an additional caveat. Moreover, we were not able to collect information regarding positive life events in the present study and thus we mainly explored the impact of negative ones. Additionally, by setting a P value of ≤.05, some significant differences in our univariate analyses may lead to false-positive findings due to multiple comparisons. Unfortunately, we were not able to apply an adjustment for multiple comparisons (e.g., Bonferroni’s adjustment)

Furthermore, we mainly analyzed a specific list of SLE: maltreatment and violence; loss events; intrafamilial problems; school and interpersonal problems based on our clinical experience as well as according to previously published studies (3, 4) without considering other types of SLE such as specific interpersonal stressors that may influence the onset/maintenance of depression in specific sociocultural environments (39).

Additionally, we did not take into account the possible link between the time from the occurrence of these events and recent SLE. For instance, one can inquire that having experienced the loss of a loved one by suicide even in the past may have influenced the likelihood to experience recent SLE. Although we did not find in our sample any recent loss event directly related to the loss of a loved one by suicide, the association between family history of suicide in the past and SLE may be affected by this caveat. Furthermore, we were not able in this study to investigate the effect of previous SLE, such as childhood traumatic experiences. Moreover, psychological factors which may further enhance the risk of negative outcome such as neuroticism, that were comprehensively examined in existing studies (19), were not considered in our study. Also, the role of potential protective factors like social support (51) or coping skills (52) were not taken specifically into account. Finally, while previous studies (53) supported a significant dose-response effect of the number of life events on illness course and outcome, here we were not able to identify the exact number of SLE.

However, despite the mentioned shortcomings, the recruitment of a large homogeneous sample of outpatients with MDD who were clearly euthymic together with the investigation of the effects of a comprehensive set of potential socio-demographic and clinical variables on the outcome need to be considered as major strengths of this study.

## Conclusion

Having a family history of suicide and history of psychotropic medications use but even the reduced use of antidepressant drugs, of which some (e.g., duloxetine) act as inhibitors of both norepinephrine and serotonin transporters (54) and others (e.g., agomelatine) are of particular interest due to their ability to enhance neuroplasticity mechanisms (55), may reflect a specific “at risk” clinical profile characterized by the enhanced vulnerability to experience SLE. This finding is in line with the expression of a complement of genes that correlates with the degree of adversity, based on the assumption that early adversities may predispose selective molecular systems and related gene expression profiles associated with impaired stress response and/or differences in the sensitivity of specific genes involved in the response to stress signals (56, 57). Further additional studies are needed in order to test these exploratory findings.

## Data Availability Statement

The original contributions presented in the study are included in the article/supplementary material; further inquiries can be directed to the corresponding author.

## Ethics Statement

The studies involving human participants were reviewed and approved by Local Ethical Review Board of the University of Genoa, Liguria, Italy. The patients/participants provided their written informed consent to participate in this study.

## Author Contributions

GS designed the study and wrote the manuscript. GC managed the literature searches and analyses. XG and PG contributed to write the first draft of the manuscript. MA provided the intellectual impetuous and MP supervised the study design and search strategy. All authors contributed to the article and approved the submitted version.

## Conflict of Interest

The authors declare that the research was conducted in the absence of any commercial or financial relationships that could be construed as a potential conflict of interest.
